# General Deficit in Inhibitory Control of Excessive Smartphone Users: Evidence from an Event-Related Potential Study

**DOI:** 10.3389/fpsyg.2016.00511

**Published:** 2016-04-14

**Authors:** Jingwei Chen, Yunsi Liang, Chunmiao Mai, Xiyun Zhong, Chen Qu

**Affiliations:** ^1^Psychology Research Center, School of Psychology, South China Normal UniversityGuangzhou, China; ^2^College of Applied Science and Technology, Hainan UniversityHainan, China; ^3^Center for Studies of Psychological Application, South China Normal UniversityGuangzhou, China; ^4^Guangdong Key Laboratory of Mental Health and Cognitive Science, South China Normal UniversityGuangzhou, China; ^5^School of Economics and Management and Scientific Laboratory of Economics Behaviors, South China Normal UniversityGuangzhou, China

**Keywords:** smartphone overuse, response inhibition, cue-related, Go/NoGo task, event-related potentials

## Abstract

With the popularity of smartphones, the problem of excessive use has drawn increasing attention. However, it is not clear whether there is an inhibitory deficit in excessive smartphone users. Using a modified Go/NoGo task with three types of context (blank, neutral, and smartphone-related), the present study combined measures of behavior and electrophysiology [event-related potentials (ERPs)] to examine general and specific inhibitory control in an excessive smartphone use group and a normal use group. Results showed that participants in both groups had larger amplitude of N2 and P3 on NoGo trials than Go trials. NoGo N2, an ERP component associated with inhibitory control, was more negative in the excessive smartphone use group than the normal use group. These results suggest that in the early stage of inhibition processing, excessive smartphone users experience more conflicts and show a general deficit that does not depend on smartphone-related cues. Moreover, the study provides further neuroscience evidence of the physiological correlates of excessive smartphone use.

## Introduction

Smartphones have significantly changed the way we live (Oulasvirta et al., 2012). People use them on a daily basis and for various purposes. It is an undeniable fact that smartphones bring many benefits. However, as they have become indispensable, severe problems have developed, for example smartphone addiction and smartphone overuse. Many people report that they cannot help using the smartphone at inappropriate moments, feel uneasy when they have to turn off their phones and have limited control of phone use (Oulasvirta et al., 2012; Lee et al., 2014). Smartphone overuse conforms to the addiction standard to some extent (Kwon et al., 2013; Lin et al., 2014).

Different from Internet overuse, which significantly correlates with the playing of computer games (Yang and Tung, 2007), smartphone overuse may be more likely to focuse on chatting and surfing the net. Compared to a computer, a smartphone is much more portable and gives people a wide variety of options, ranging from calling to navigation, playing games, and social networking. It provides convenient access to a large amount of online content and opportunities to maintain social relationships, involving more and more people. Additionally, the smartphone is approachable and gives users instant gratification, reinforcing continuous usage (Oulasvirta et al., 2012). The Korea National Information Society Agency (NIA) reported that the group that was labeled as showing ‘smartphone addiction’ has exceeded Internet addiction (7.8%) since 2011, which indicated that smartphone overuse may be a more serious problem than Internet addiction.

Smartphone overuse includes some features of Internet addiction, such as prominence, mood modification, tolerance, withdrawal, conflict, and relapse (Van Rooij et al., 2010; Weinstein and Lejoyeux, 2010; Kwon et al., 2013; Lin et al., 2014). Billieux (2012) suggested that problematic use of the mobile phone should be viewed as a disorder and conceptualized as an addictive behavior. In this respect there may be similarities in the inhibitory control problems that are seen in various forms of addictive behaviors. Heavy drinkers have been shown to make more mistakes in the Stroop task (Field et al., 2007) and Go/NoGo task (Petit et al., 2012). Likewise, smokers show worse behavioral inhibition control than non-smokers (Spinella, 2002; Luijten et al., 2011). Littel et al. (2012) and Liu et al. (2014) also found that excessive gamers had worse inhibition control compared to other gamers. In addition, Dong et al. (2010) suggested that Internet addicts showed lower activation in the record of event-related potential (ERP) in the early conflict detection stage, which made them need to put in more effort to finish the behavioral inhibition task in the late stage. However, to our knowledge there have been no empirical studies conducted on inhibition control in smartphone excessive users. Therefore, the purpose of the present study was to test the hypothesis that excessive smartphone users show deficits in inhibitory control.

To investigate the inhibitory control in addictive individuals, researchers to date have used the Go/NoGo task (Spinella, 2002; Dinn et al., 2004; Monterosso et al., 2005; Reynolds et al., 2007; Luijten et al., 2011). The recording of electroencephalographic (EEG) activity has been suggested to be a more sensitive index of response inhibition. There are two major ERP components associated with response inhibition. One is NoGo N2, which is an enhanced negative wave. It presents approximately 200–300 ms after the emergence of the NoGo stimulus and is maximal in the prefrontal lobe. Several studies have indicated that the amplitude of NoGo N2 correlates with behavioral data showing inhibition control (Falkenstein et al., 2000; Nakata et al., 2004; Kaiser et al., 2006). In these studies, the more difficult the task was, the higher the amplitude of N2, suggesting that the amplitude of N2 reflects inhibition control. NSSI (Lixia et al., 2013) found larger amplitude of NoGo N2, which indicated that participants found it more difficult than control group to finish the same task and had deficits in executive inhibitory functions. Another ERP component associated with response inhibition is NoGo P3, which presents approximately 300–500 ms after stimulus emergence. Dimoska et al. (2006) suggested that P3 may reflect an inhibition process in or near the motor or premotor cortices in the late stage.

Although, there have been many studies on the inhibition control of addicts that have adopted the Go/NoGo paradigm, there have been inconsistent results across studies. Dong et al. (2010) adopted the typical Go/NoGo task and ERP technique to investigate the relationship between what was termed Internet addiction and conflict detection ability. The results showed that the NoGo N2 of Internet addicts was lower than the control group and the NoGo P3 was higher and had a longer peak latency.Littel et al. (2012) also used the Go/NoGo task to investigate the inhibition of excessive computer gamers, but they did not find ERP differences in NoGo trials.

In addition, some researchers have also pointed out that there may be a reciprocal relation between the processes of executive functioning and the activation of conditioned drug-related stimuli. It is said that the drug-related stimuli are more attractive to addictive individuals, which may cause more severe inhibition control problems (Dawe et al., 2004; Olmstead, 2006). Petit et al. (2012) adopted ERP to investigate the effect of alcohol-related cues on the inhibition control of social drinkers under the Go/NoGo task using a block design, and found that heavy drinkers made more mistakes and had longer peak latency of NoGo P3 only in the alcohol-related context. Brand et al. (2014) suggested that there was a strong relationship between Internet-related stimuli and positive or negative reinforcement. This conditioned relationship makes us increasingly harder to cognitively control the Internet use, even though we realized that the Internet overuse may be related to negative consequences in the long run. As a result of classical and instrumental conditioning processes (Everitt and Robbins, 2005), the nucleus accumbens and parts of the dorsal striatum together with limbic and para-limbic regions (e.g., the orbitofrontal cortex) learn to habitually react on drug cues with craving and the dorsolateral prefrontal cortex, which is linked to higher-order cognitive functions, loses its regulatory influences (Bechara, 2005; Goldstein et al., 2009). This is most likely the consequence of changes in the dopaminergic reward system by frontal-guided changes of glutaminergic innervation of the nucleus accumbens and related brain areas (Kalivas and Volkow, 2005).

However, Luijten et al. (2011) also used a modified Go/NoGo task and ERP technique to investigate the inhibition control of smokers. The smokers had worse behavioral inhibition control and reduced amplitude of NoGo N2 than the control group. However, there existed no difference based on the picture content of the stimulus (smoking versus neutral). Specifically, no addicted cue-related effect was found. This inconsistent result was suggested to be due to the research design, which presented different types of pictures (some smoking-related, some not) in one block. The addicts may be affected by the smoking-related pictures, which may evoke their craving before the task performance. As a result, the present study adopted the blocked design to prevent interference between the type of pictures, and required the participant to respond to the frame of picture.

In summary, the aim of the present study was to investigate the inhibition control of smartphone excessive users by using an adapted paradigm to assess behavioral and electrophysiological responses. For this purpose, a novel Go/NoGo paradigm, with three types of context: blank, neutral, and smartphone-related pictures, was used. We examined the NoGo N2 and P3 components of the excessive smartphone users, and compared them with a control group. In addition, the present study used a cellphone application to record smartphone usage, information which was used to divide the participants into excessive users and control groups more reliably. We hypothesized that relative to controls, excessive smartphone users would have a deficit in inhibitory control, both in blank context and in the smartphone-related context (SC).

## Materials and Methods

### Participants

Three hundred and twenty students were recruited from our local university community to participate in a survey of phone usage. Data from 17 participants were excluded because more than 50% of the values were missing. From the remaining 303 participants, we included 19 participants as the excessive users group and 19 participants as the control group. All 38 participants volunteered to attend the ERP experiment and signed informed consent, and the research was approved by the Human Research Ethics Committee of South China Normal University. All participants were right-handed, reported no history of seizures, periods of unconsciousness, psychiatric illness, or uncorrected vision problems. Two participants who quit the exam halfway were excluded from behavioral analyses. Behavioral data of 18 participants (eight males, mean age: 19.56 ± 1.25) as the excessive smartphone use group and 18 participants (nine males, mean age: 19.78 ± 1.21) as the control group were analyzed. *t*-test comparison regarding age was not significant [*t*(34) = -0.54, *p* = 0.59]. In addition, four participants who had too few ERP segments of adequate quality were later excluded from ERP analyses. Finally, ERP data of 16 participants (seven males, mean age: 19.50 ± 1.27) as the excessive smartphone use group and 16 participants (nine males, mean age: 19.69 ± 1.30) as the control group were analyzed.

### Manipulation Check

#### Smartphone Addiction

The Smartphone Addiction Inventory (SPAI; Lin et al., 2014) was used to measure smartphone usage. This scale contains four factors of problematic smartphone usage (compulsive behavior, functional impairment, withdrawal, and tolerance). The SPAI yields a total score that is indicative of the severity of smartphone addiction; higher scores indicate more severe addiction. The SPAI has 26 items that participants rate on four-point scales from 1 (strongly disagree) to 4 (strongly agree). The Cronbach’s alpha coefficient for the SPAI is 0.94 and the Cronbach’s alpha coefficient in our sample is 0.88.

**FIGURE 1:**
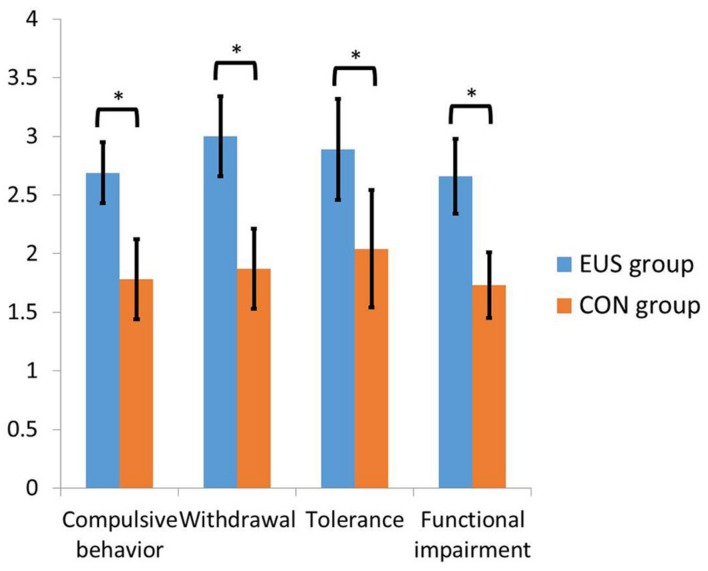
**The score of four factors (compulsive behavior, functional impairment, withdrawal and tolerance) in SPAI between groups.** Error bars represent standard deviation (SD). *Significant difference refers to a *p* < 0.05.

We defined the upper 30% of SPAI scores (62.46 or higher) as the excessive smartphone (ESU) use group and the lower 30% of SPAI scores (56 or lower) as the control group. **Table 1** presents the difference between the score of the final sample of smartphone overuse and control groups and **Figure 1** show the scores and standard deviation (SD) of four factors (compulsive behavior, functional impairment, withdrawal and tolerance) in SPAI between groups.

**Table 1:** The SPAI score between groups.

	EUS group			CON group				Between group comparision *p*-value
	Mean (SD)	Maximum	Minimum	Mean (SD)	Maximum	Minimum	*t*	
Compulsive behavior	2.69 (0.26)	3.11	2.11	1.78 (0.34)	2.22	1.22	10.04	0.0001
Withdrawal	3.00 (0.34)	3.67	2.17	1.87 (0.34)	2.50	1.17	10.10	0.0001
Tolerance	2.89 (0.43)	3.67	2.33	2.04 (0.50)	2.67	1.00	5.51	0.001
Functional impairment	2.66 (0.32)	3.25	1.75	1.73 (0.28)	2.25	1.38	9.35	0.0001
Total	72.22 (6.00)	85	63	47.17 (5.61)	56	33	12.95	0.0001

#### Data Collection of Smartphone Usage

App Timer Mini 2 Pro, an Android phone app developed by KF Software House, is a time management tool for managing and analyzing the user’s time spent on apps. It keeps track of detailed information about the use of selected apps, including when and how many times selected apps are used, the amount of time spent on them and also data of touching phone screen. In the present study, we adopted the app to unobtrusively collect data from participants’ own smartphones.

**FIGURE 2:**
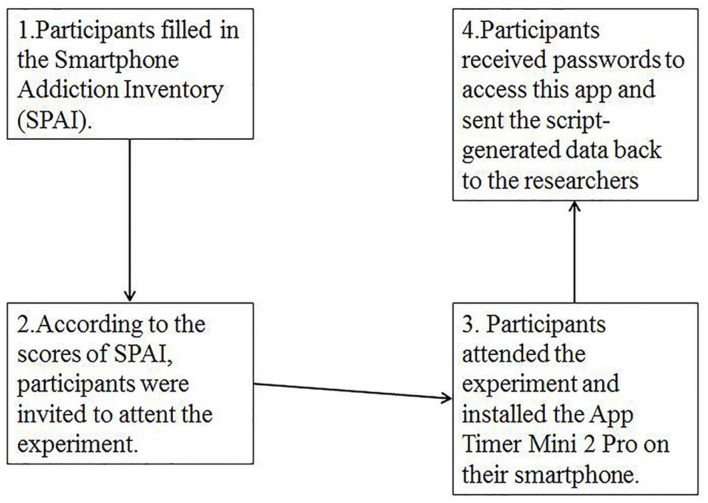
**The flowchart depicting the participants selection**.

Thirty six students, who met the inclusion criteria of SPAI, were asked to install App Timer Mini 2 Pro and select 20 frequently used apps on their smartphones to be tracked for 1 week. This app was encrypted to prevent unintentional or intentional viewing or modifying of the information being tracked by the app. A week later, participants received passwords to access this app and sent the script-generated data back to the researchers. Data from one participant were excluded because he quit halfway. The smartphone excessive use group showed significantly more frequent use [*t*(33) = 3.59, *p* < 0.001] and more time spent using the smartphone [*t*(33) = 3.33, *p* < 0.001] compared to the control group. Also, the control group used a wider variety of apps while the excessive smartphone users mostly used social media app like WeChat and QQ. The manipulation check process is shown in **Figure 2**.

### Task Paradigm

The stimuli consisted of three types of picture (189*189 pixels): blank, neutral, and smartphone-related. Each picture was inside a frame, to be clearly visible against the background. According to some studies, the fast growth in use of online social networking services (SNSs) may be the main reason for smartphone overuse (Salehan and Negahban, 2013; Wu et al., 2013); as a result, we adopted social media icons as the smartphone-related pictures. 100 pictures were selected from screenshots of the QQ and WeChat application and 100 pictures were selected from the National Affective Picture System (Lang et al., 1997) and Chinese Affective Picture System (Lu et al., 2005). Then, 32 students who did not take part in the ERP study rated these pictures for emotional level. Students were asked to rate how pleasant the picture was on a scale from zero (very unpleasant) to nine (very pleasant). Finally, 50 neutral pictures were selected as neutral background (mean score = 4.87 ± 0.50), 25 WeChat pictures were selected for WeChat background (mean score = 4.75 ± 0.13), and 25 QQ pictures were selected for QQ background (mean score = 4.89 ± 0.19). All 100 pictures have neutral emotional rating.

**FIGURE 3:**
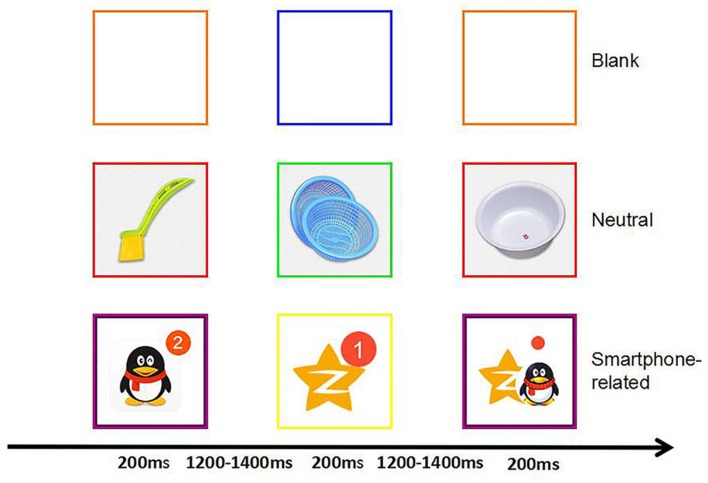
**Trial design for a modified Go/NoGo task.** In this task, participants were presented with six blocks of 250 trials, divided in 200 Go trials and 50 NoGo trials.

A modified Go/no-go task was developed for the purposes of the current study. All participants were presented with a series of pictorial stimuli with six kinds of frame (red and green, orange and blue, purple and yellow), and asked to respond or inhibit response according to the color of the picture frame. That is, the frame color prompted whether a stimulus was a Go or a NoGo trial. For example, the red color indicated that the participant should press the button as fast as possible (Go trial) and the green indicated no press (NoGo trial). 20% of all trials were NoGo trials. Each picture was presented 10 times during the whole task, eight times as a Go stimulus, and two times as a NoGo stimulus. First, participants were given the opportunity to practice in 15 trials before each block. Then the actual task was started. At the beginning of each trial, a red fixation was displayed for 1000 ms to center concentration on the task. Each picture was displaced for 200 ms, followed by a return to the initial gray screen (1200–1400 ms). Thus, subjects had a maximum of 1400–1600 ms to press the button before the next picture appeared (see **Figure 3** for an example of a smartphone-related trial). Participants were asked to look at the center of the screen continuously and to refrain from moving and blinking during blocks to reduce interference caused by movements. Between blocks, participants received a break.

The formal experiment consisted of six blocks of 250 trials per block, with two blocks for each type of context. Go and No-Go trials were displayed in a semi-random order to avoid the consecutive presentation of two NoGo trials within the same block. The two frame colors of each block were different from each other and the sequence of blocks was counterbalanced among participants.

### Procedure

Each participant was seated alone in a comfortable EEG chair in a light and sound-attenuated room approximately 75 cm from a computer screen with the horizontal and visual angles below 5°. Electrodes were attached and task instructions were explained. Participants performed the Go/NoGo tasks during EEG recording. Participants were paid 40 yuan as basic payment and informed before the task that the six best-performing (shorter reaction time, accuracy rate ≥ 90%) participants would be rewarded with additional payment (5 yuan).

### ERP Recording

The EEG was recorded using the Scan software (Neuroscan, 4.5) from 32 scalp sites (positioned following the International System in an elastic cap). The vertical electrooculograms (VEOGs) were monitored with electrodes located above and below the left eye. The horizontal (HEOG) was recorded by electrodes placed 1.5 cm lateral to the left and right external canthi. All signals were digitalized with a sample rate of 500 Hz and A/C conversion with a bandpass of 0.01–100 Hz. The interelectrode impedances were maintained below 5 kΩ. Data were segmented in epochs of 1 s (200 ms before and 800 ms after response or stimulus presentations). After ocular correction epochs including an EEG signal exceeding ±80 μV were excluded from the average. The mean 200 ms pre-response or pre-stimulus period served as baseline. After baseline correction, average ERP waves were calculated for artifact-free trials at each scalp site for correct and incorrect responses separately. Segments with incorrect responses (miss for Go trials or false alarm for NoGo trials) were excluded from EEG analyses.

### Statistical Analysis

The design was a three factor mixed design with the first factor referring to the type of context (blank, neutral, and smartphone-related), the second factor referring to the trial type (Go, NoGo) and the third factor referring to the group (excessive smartphone user, normal user). The N2 was the most negative value within the 220–320 ms time interval after stimulus onset and was studied at a cluster of frontocentral electrodes, including Fz and FCz. The P3 was the most positive value within the 350–500 ms time interval after stimulus onset. The P3 was studied at a cluster of central electrodes, including CPz and Pz. And since the number of trials in Go and No Go conditions was not equal (4:1), we selected only one quarter of the Go trials randomly for further comparison.

All analyses were performed using SPSS 18.0 and significance level was set at *p* = 0.05. Repeated Measures Analyses of Variance (RM-ANOVA; with Greenhouse-Geisser adjusted *p*-values) were applied to analyze behavioral outcomes of performance on the Go/NoGo task, as well as ERP as the index of response inhibition. Simple effects were explored and interaction sources were systematically examined. A Bonferroni correction for multiple comparisons was applied in all *post hoc* analyses.

## Results

### Performance Measures

The response accuracy were presented in **Table 2**. We computed a three-factor repeated measures analysis of variance on the accuracy rates as the dependent variable, groups as the between factor, and context as the repeated measure. The accuracy rates were calculated separately for the task context (blank, neutral, and smartphone-related), for trial type (Go and NoGo) for every subject in both groups. This yielded a main effect of trial type [*F*(1,34) = 99.77, *p* < 0.001, $\eta_{p}^{2}$ = 0.75], which indicated that accuracy was higher on the Go trials in both groups. There was also an interaction between trial type and context [*F*(1.40,47.56) = 8.86, *p* < 0.01, $\eta_{p}^{2}$ = 0.21]. On Go trials, the main effect of context was not significant in either group. However, on NoGo trials, there was a main effect of context [*F*(2,33) = 18.74, *p* < 0.001, $\eta_{p}^{2}$ = 0.53]. Multiple comparisons revealed that participants showed higher accuracy in the blank context than in the neutral [*t*(35) = 5.37, *p* < 0.001, *d* = 0.23] and smartphone-related [*t*(35) = 3.56, *p* = 0.001, *d* = 0.39] contexts. No main or interaction effects of group were found for accuracy rate.

**Table 2:** Accuracy of the participants (M ± SD).

	Blank context		Neutral context		Smartphone-related context	
	Go	NoGo	Go	NoGo	Go	NoGo
ESU group	0.97 ± 0.03	0.79 ± 0.17	0.96 ± 0.04	0.73 ± 0.19	0.97 ± 0.05	0.74 ± 0.17
CON group	0.99 ± 0.01	0.85 ± 0.08	0.99 ± 0.01	0.81 ± 0.09	0.98 ± 0.02	0.80 ± 0.15

The same three-factor repeated measures analysis of variance was used to analyze the reaction time data, and a significant main effect of context was found, *F*(1.76,59.94) = 6.65, *p* < 0.005. Multiple comparisons revealed that participants generally responded faster to the blank context than neutral [*t*(35) = -3.33, *p* < 0.005, *d* = -0.24] and smartphone-related [*t*(35) = -3.64, *p* < 0.001, *d* = -0.25] contexts. No other significant effects were found for reaction times.

### ERP Results

**FIGURE 4:**
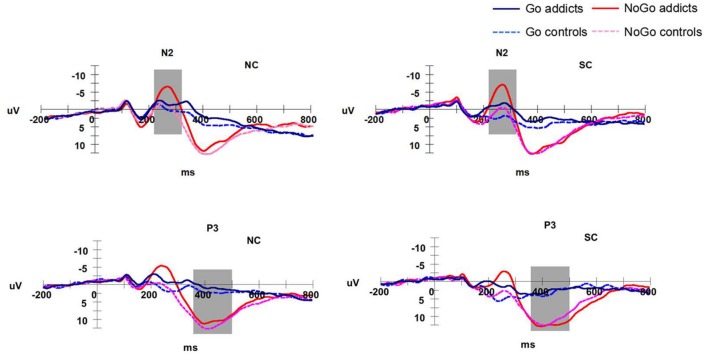
**The ERP waveforms at FCz and CPz electrode sites**.

The N2 and P3 amplitude for neutral context (NC) SC of both groups in Go/NoGo task is displayed in **Figure 4**.

#### N2 Amplitudes

**FIGURE 5:**
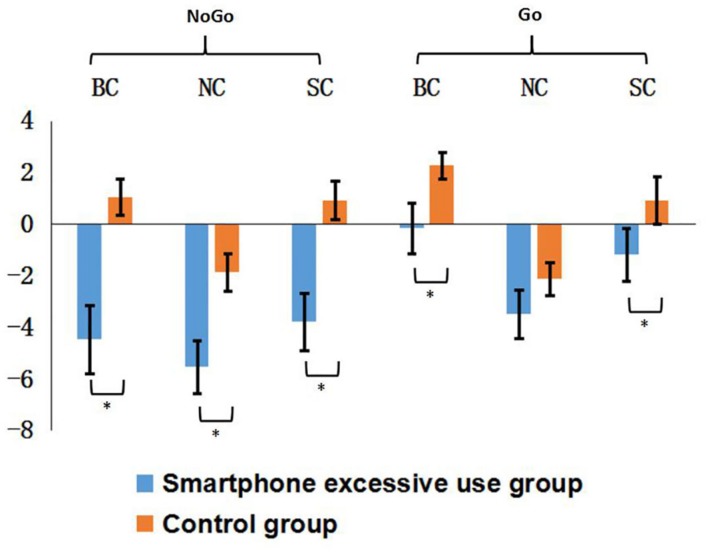
**Mean amplitude of N2 for smartphone excessive group and control group in different context.** Error bars represent standard error (SE). N2 amplitude recorded in Fz and Fcz channels. *Significant difference refers to a *p* < 0.05.

A robust main effect of trial type was found, *F*(1,30) = 16.93, *p* < 0.001, $\eta_{p}^{2}$ = 0.36 showing that the mean amplitude of N2 was larger for the NoGo than Go condition. There was also a main effect for Group [*F*(1,30) = 11.67, *p* < 0.005, $\eta_{p}^{2}$ = 0.28], which indicated that N2 mean amplitude was larger in the excessive smartphone use group than control group. Also, the main effect of context was significant [*F*(2,60) = 25.11, *p* < 0.001, $\eta_{p}^{2}$ = 0.46]. Importantly, a group*trial type interaction effect was found [*F*(1,30) = 6.21, *p* < 0.005, $\eta_{p}^{2}$ = 0.18]. N2 mean amplitude was larger in the excessive smartphone use group than control group on NoGo trials. Further analysis indicated a significant difference between the smartphone overuse and normal user groups in the NoGo condition; the excessive smartphone use group elicited significantly larger N2 mean amplitude than the normal users group in blank [*F*(1,30) = 13.57, *p* = 0.001, $\eta_{p}^{2}$ = 0.31], neutral [*F*(1,30) = 8.67, *p* = 0.006, $\eta_{p}^{2}$ = 0.22], and smartphone-related [*F*(1,30) = 12.40, *p* = 0.001, $\eta_{p}^{2}$ = 0.30] contexts. Moreover, there was a significant interaction between context and trial type [*F*(2,60) = 4.61, *p* < 0.05, $\eta_{p}^{2}$ = 0.13]. The mean N2 amplitude was largest in the NC. The interaction of group and context was not significant in N2 mean amplitude [*F*(2,60) = 1.66, *p* = 0.199, $\eta_{p}^{2}$ = 0.52]. Neither was the triple interaction [*F*(2,60) = 2.97, *p* = 0.384, $\eta_{p}^{2}$ = 0.03]. The results are illustrated in **Figure 5**.

#### P3 Amplitudes

The significant main effect of trial type indicated P3 amplitudes were larger for the NoGo than the Go condition [*F*(1,30) = 153.83, *p* < 0.001, $\eta_{p}^{2}$ = 0.86]. Also, there was a main effect of context [*F*(2,60) = 4.94, *p* = 0.01, $\eta_{p}^{2}$ = 0.14]. Multiple comparisons revealed that the blank context had significantly higher P3 amplitude than the NC [*F*(1,31) = 9.74, *p* < 0.005, $\eta_{p}^{2}$ = 0.24]. Moreover, there was a significant triple interaction [*F*(2,60) = 3.22, *p* < 0.05, $\eta_{p}^{2}$ = 0.97]. Further analysis showed that the blank context had significantly higher P3 amplitude than the NC on Go items in excessive smartphone overuse group [*F*(1,15) = 7.876, *p* < 0.05, $\eta_{p}^{2}$ = 0.34]. No main effect for group and no interaction effects were found for the P3 component.

Besides, neither behavioral performance nor ERP performance has significant correlation with the use pattern written by the app.

## Discussion

Using ERP technique, the present study investigated general response inhibition and specific response inhibition in response to smartphone-related stimuli in excessive smartphone users using a modified Go/NoGo paradigm. At the neural level, N2 amplitudes related to NoGo trials were more negative than N2 related to Go trials. Moreover, as predicted, excessive smartphone users showed larger NoGo-N2 compared to controls, indicating that the excessive smartphone users had general deficits in the early stage of inhibitory control.

On the behavioral level, no differences between groups were found on accuracy or reaction time. We may conclude that the deficit may not appear on the behavior level, but may still exist on the electrophysiological level. Some studies have proposed that NoGo N2 might reflect conflict monitoring, and the high frequency of go signals could elicit a larger N2 (Nieuwenhuis et al., 2003; Donkers and van Boxtel, 2004; Randall and Smith, 2011). Other studies have suggested that the NoGo N2 reflects inhibitory neural processes (Jodo and Kayama, 1992; Falkenstein et al., 1999). People with unusually high impulsivity may have significantly different amplitude of N2. For example, Lixia et al. (2013) found people with Non-Suicidal Self-Injury (NSSI) had larger NoGo N2 than controls. Similar larger amplitude of N2 existed for children with ADHD in the Eriksen flanker task. In the present study, excessive smartphone users’ N2 amplitudes in all NoGo trials (20% of all trials) were larger than the N2 in Go trials (80% of all trials), which is consistent with “conflict monitoring” and “inhibitory process” theory. Besides, it has been indicated that the ACC, a neural generator of N2 (Nieuwenhuis et al., 2003; Bekker et al., 2005; Jonkman et al., 2007), acts to evaluate response conflicts and upregulate cognitive control (Botvinick et al., 2001; Carter and Van Veen, 2007). As such, an increase in N2 amplitude (or enhanced ACC activation) seen in the present study may reflect enhanced conflict due to the upregulation of cognitive control, especially inhibition. Based on these assumptions, the present finding of an enhanced pattern of N2 amplitudes in smartphone excessive users but similar behavior accuracy relative to controls, implies that in order to achieve similar behavioral performance, excessive smartphone users have to make more effort. This indicates that when the excessive smartphone users face the NoGo signal, they might not be able to flexibly upregulate cognitive control. To meet the task demands, they may require greater cognitive control compared to the control group. If so, excessive smartphone users might have deficits in the early stage of inhibitory control. This result is consistent with the research of cognitive control in media multitaskers (Ophir et al., 2009). As the smartphone can be used to perform different task simultaneously, they may be more susceptible to interference from irrelevant stimuli and find it more difficult to suppress the activation of irrelevant task sets like, because they need to sacrifice performance on the primary task to let in other sources of information. And it can also be explained by control processes in developing and maintaining an addictive use of the Internet. Brand et al. (2014) hypothesized the expectancies about what the Internet can provide and what a person may expect from using the Internet may be in a conflict with the individual’s expectancies about potential negative consequences in the short or the long run, which are associated with an Internet overuse.

Enlarged amplitudes of NoGo P3 relative to Go P3 were observed, whereas there existed no difference in NoGo P3 amplitude between groups and contexts in the present study. Some researchers have suggested that NoGo P3 is correlated with the inhibition process in the late stage (Dimoska et al., 2006) and the actual inhibition of the motor system (Kok et al., 2004). As excessive smartphone users often successfully engage in multitasking with their electronic devices, like watching TV while talking and texting on phone, their executive capacity may not differ from normal smartphone users. As a result, excessive smartphone users in our study may not have deficits in the late stage of inhibition.

Another aim of the present study was to explore inhibitory control in an addiction cue-related context. However, although we adopted the blocked design, the SC did not elicit different responses from the neutral or blank contexts, either on the behavioral or electrophysiological level. Indeed, previous studies did not find consistent results in terms of whether addiction-related cues affected inhibition control. Some studies on addiction have shown that addicts have a preference for drug-related cues (Petit et al., 2012) while other studies like Luijten et al. (2011) showed no difference among contexts. As a result, we believe that the inhibition deficit of excessive smartphone users may be general and not affected by the smartphone-related cue. Another interpretation is that the inconsistent result may be related to the varying degree of addiction (Franken, 2003), as well as defective paradigms. For example, Mogg et al. (2005) found that smokers with lower levels of nicotine dependence had higher levels of craving in response to smoking-related pictures as reflected by greater maintained attention. Maurage et al. (2014) found that alcohol-dependent individuals presented globally delayed reaction times compared to controls which did not depend on cue. With a preserved performance for alerting and orienting networks, they showed impaired executive control. This deficit was positively correlated with the duration of alcohol-dependence habits, the number of previous detoxification treatments and the mean alcohol consumption before detoxification. This result may be explained by the integrated “incentive-habit” theories of addiction (Di Chiara, 2000), which suggest that the effect of incentive motivational processes on behavior diminishes in strength as addiction progresses, due to a switch from “incentive responding” to “habit responding.” In addition, Davis (2001) introduced a theoretical cognitive–behavioral model on pathological or problematic Internet use and differentiates between a generalized pathological Internet use, which we call generalized Internet addiction (GIA), and a specific pathological Internet use, for which we use the term specific Internet addiction (SIA). Consequently, it is assumed that GIA is directly linked to the options the Internet itself provides, while SIA can also be developed outside the Internet, but is aggravated by the enormous functions offered by the Internet applications (Brand et al., 2014). From this point, we can also assume that smartphone overuse was a generalized smartphone addiction. The current study is the first to investigate the inhibitory deficit of excessive smartphone users. Different from internet addiction, the deficit that smartphone overuse brought was more popular and perceptually invisible. Excessive smartphone users showed a general deficit in inhibitory control in the early stage of information processing, unrelated to smartphone-related stimuli. This finding makes a unique contribution to the literature. As Billieux et al. (2015) pointed out that most studies which conducted to identify new behavioral addictions failed to consider two factors that are in their view mandatory to define a pathological condition, namely functional impairment and stability of the dysfunctional behavior, the present study provides evidence to define excessive smartphone overuse as a kind of behavioral addition, for excessive smartphone users have deficit in inhibitory control, which was a kind of functional impairment. On the other hand, this study clearly presents some limitations. We must acknowledge the fact that the control group still used smartphones in their daily lives. The difference between the excessive smartphone use group and the control group was not as large as in research on drug addiction and the approach of distinction merely based on criteria, which should shift toward an approach focusing on the processes involved (Billieux et al., 2015). And no significant correlations of behavioral or ERP performance with the use patterns written by the app was found, which might suggest that people who use more smartphone but not be addicted with it may not have deficit in inhibitory control. Further evidence is expected to tackle this point. Besides, the present study could not conclude a causality between smartphone overuse and inhibitory control. To approach such a complex causality, longitudinal studies, experimental manipulation, and more sophisticated statistical methods could be used in the future (Maurage et al., 2013). And our future research is to investigate the inhibitory control on excessive smartphone users in the stage of stopping using smartphone.

## Conclusion

The present study demonstrated that in the early stage of inhibition processing, excessive smartphone users experienced more conflicts than the control group and showed a general deficit in the early stage of inhibition processing. ERP results indicated that the amplitudes of NoGo N2 were larger than the amplitudes of Go N2 and the excessive use group had larger amplitudes of NoGo N2. In addition, the SC relative to blank and NCs showed no significant difference on either the behavioral or electrophysiological level, suggesting that the deficit seen in excessive smartphone was general and not dependent on the presence of smartphone-related cues.

## Author Contributions

Prof. QU and JC designed the experiment. JC, CM, and XZ performed the experiment. JC and YL processed the data. Manuscript was written by JC and YL and edited by Prof. QU.

## Conflict of Interest Statement

The authors declare that the research was conducted in the absence of any commercial or financial relationships that could be construed as a potential conflict of interest.
